# Future perspectives for advancing regulatory science of nanotechnology-enabled health products

**DOI:** 10.1007/s13346-022-01165-y

**Published:** 2022-06-12

**Authors:** Blanka Halamoda-Kenzaoui, Robert Geertsma, Joost Pouw, Adriele Prina-Mello, Moreno Carrer, Matthias Roesslein, Adrienne Sips, Klaus Michael Weltring, Kathleen Spring, Susanne Bremer-Hoffmann

**Affiliations:** 1 European Commission Joint Research Centre (JRC), Ispra, Italy; 2 Centre for Health Protection, National Institute for Public Health & the Environment (RIVM), Bilthoven, The Netherlands; 3 Laboratory for Biological Characterization of Advanced Materials (LBCAM), Trinity Translational Medicine Institute (TTMI), Trinity College Dublin, St James’s Hospital, Dublin 8, Ireland; 4 Nanomedicine and Molecular Imaging Group, Clinical Medicine, Trinity Translational Medicine Institute (TTMI), Trinity College Dublin, St James’s Hospital, Dublin 8, Ireland; 5 Trinity St James’s Cancer Institute, Trinity College Dublin, St James’s Hospital, Dublin 8, Ireland; 6 Swiss Federal Laboratories for Material Science and Technology, EMPA, St. Gallen, Switzerland; 7 Centre for Safety of Substances and Products, National Institute for Public Health & the Environment (RIVM), Bilthoven, The Netherlands; 8 Gesellschaft Für Bioanalytik Muenster E.V, Muenster, Germany

**Keywords:** Nanomedicine, Nanomedical devices, Regulation, Method standardisation, Harmonisation

## Abstract

The identification of regulatory challenges for nanotechnology-enabled health products, followed by discussions with the involved stakeholders, is the first step towards a strategic planning of how such challenges can be successfully addressed in the future. In order to better understand whether the identified regulatory needs are sector-specific for health products or might also hinder the progress in other domains, the REFINE consortium reached out to communities representing other sectors that also exploit the potential of nanotechnology, i.e. industrial chemicals, food and cosmetics. Through a series of trans-sectorial workshops, REFINE partners identified common as well as sector-specific challenges and discussed possible ways forward. Potential solutions lie in a more strengthen collaboration between regulatory and research communities resulting in a targeted production and exploitation of academic data for the regulatory decision-making. Furthermore, a coordinated use of knowledge sharing platforms and databases, trans-sectorial standardisation activities and harmonisation of regulatory activities between geographical regions are possible ways forward, in line with the upcoming European political initiatives such as the Chemical Strategy for Sustainability (CSS). Finally, we also discuss the perspectives for further development and sustainability of methods and tools developed in the REFINE project.

**Graphical abstract:**

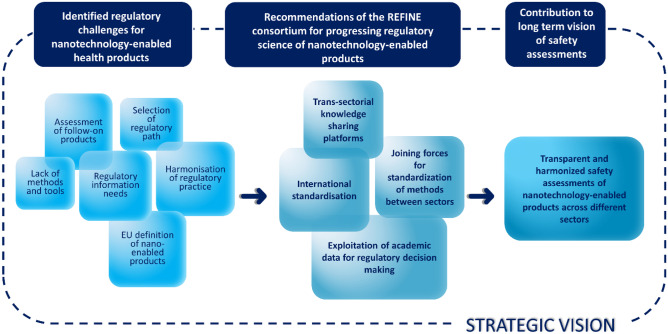

## Introduction

Nanotechnology-enabled health products can offer exciting possibilities for diagnostic and therapeutic tools, but their regulation including proper quality and safety assessments might be challenging, due to their specific properties and complex structure. Understanding of such regulatory challenges and the development of adequate methodological tools and approaches was among the goals of the Horizon 2020 project REFINE (Box 1). Identified regulatory needs for nanotechnology-enabled health products were summarised in the REFINE White Paper [1] early in the course of the project (Table 1). It allowed guiding of the experimental work in REFINE but also engaging with various stakeholder communities to raise awareness and propose potential solutions to the identified needs. In addition to publishing the work in a scientific journal [2], the project partners regularly presented results at scientific conferences and dedicated workshops, as well as to different working groups of international and European competent authorities^1^,^2^,^3^. In this way, the REFINE consortium initiated a dialogue with the scientific community, industry representatives, policy makers and regulatory scientists. These activities were complemented with an online survey and interactive sessions at the Knowledge Exchange Conferences (KECs), organised as part of the REFINE work programme and provided quantitative feedback on selected questions. Such regular stakeholder engagement allowed to obtain a better overview on the relevance of the identified regulatory challenges, as perceived by the different communities, and to understand whether additional, more specific, challenges and needs are present.

**Table 1:** Short description and examples of regulatory challenges for nanotechnology-enabled health products, identified in the REFINE White Paper [1]

**Challenge**	**Description**	**Specific examples**
**Identification of regulatory information needs specific for nanotechnology-enabled products**	It is not completely clear yet which nano-specific parameters have an impact on safety and efficacy of the product and should be characterised in the regulatory assessment	For some types of nanotechnology-based platforms, the specific guidance is lacking, and regulatory information requirements are not defined
**Availability of suitable test methods**	Suitable, validated (and often specific for nanotechnology-based products) test methods are needed to provide regulatory information [3]	Methods are lacking, e.g. for measurement of the surface coating heterogeneity and composition of the protein corona, resulting in additional challenges for product developers, who need to develop and validate a specific method
**Selection of the regulatory framework (“borderline products”)**	In theory, it should be clear from regulatory definitions whether a product is regulated as a medicinal product or as a medical device. In practice, nanotechnology-based products are regularly “borderline products” for which the selection of the correct regulatory framework is a challenge	Products based on iron oxide have been regulated as medicinal products, e.g. to treat iron deficiency or as MRI contrast agent, and as medical devices, e.g. to treat tumours via hyperthermia or to localise sentinel lymph nodes during surgical removal of tumours
**Assessment of the equivalence of follow-on products (“nanosimilars”)**	It is not known yet which characteristics of the follow-on products should be assessed to define its equivalence to the reference product [4]	A number of follow-on iron sucrose complexes or liposomal doxorubicin have been commercialised, but the clinical performance of different follow-on formulations has hardly been compared [5]
**Harmonisation of the regulatory practice in different regions**	Regulatory path and information requirements can differ in different countries and regions providing a challenge for drug developers seeking access to different markets	The same liposomal product was accepted as a generic product in the US but not accepted as such in the EU [6]
**Application of the classification rule (rule 19) in combination with the EU definition of nanomaterial**	Classification rule 19 in the medical device regulation determines the conformity assessment procedure to be applied for medical devices incorporating or consisting of nanomaterial(s). Interpretation of this rule and the EU definition for nanomaterial that is included in the regulation is not always straightforward when applied to actual products	Bone fillers with nanomaterials in their formulation are class III, bone fixation screws with a nano coating are class IIb, dental filling materials with nanomaterials are class IIa. Guidance on interpretation of all classification rules including rule 19 was recently published by the European Commission [7]

The opportunities provided by nanomaterials are also exploited in other sectors, and even if the risk assessment-based regulatory decisions can be very different, it became obvious that the sectors share also common challenges including the implementation of EU recommendation on the definition of nanomaterial or safety aspects related to the toxicokinetic of a material. There are certainly other commonalities but also differences which would require an in-depth analysis.

For this reason, to achieve a better understanding on common and sector-specific challenges, the consortium initiated a close exchange with its highly complementary project Gov4Nano^4^. These discussions resulted in a proposal to develop trans-sectorial strategies in order to share common burden and to provide future perspectives for a harmonised regulation of products based on nanotechnologies. The exchange of scientific knowledge on nanomaterial characterisation and safety assessments becomes even more relevant in the currently changing political environment [8] since the new Chemical Strategy for Sustainability (CSS) highlights the need for a trans-sectorial alignment of risk assessments of substances while taking into account the specificities of each sector (*one substance one assessment initiative*) [9]. The trans-sectoral KECs^5^ organised by REFINE and the Gov4Nano (Trans)Regulatory Risk Analysis Summit (RRAS)^6^, demonstrate, on the example of nanomaterials, a way forward to coherent safety assessments of chemicals across legislations.

Box 1 REFINE project Horizon 2020 project (2017–2022) Grant Agreement No. 761104 Aim: Development of a Regulatory Science Framework for Nano(bio)material-based Medical Products and Devices

Website:http://refine-nanomed.eu/

Twitter account: https://twitter.com/REFINE_H2020

REFINE consortium involved 13 European partner organisations from academia, governmental organisation and industry. It focused on addressing regulatory needs for nanotechnology-enabled health products, optimisation and validation of characterisation methods, development of a decision support system and engaging with stakeholders from health and other sectors

## Engagement with stakeholder communities from different industrial sectors

**Fig. 1:**
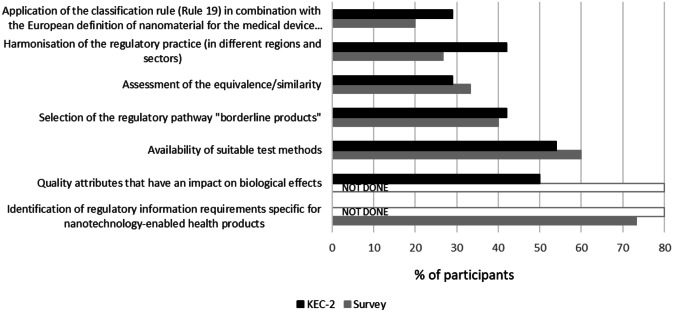
Relevance of the regulatory challenges for nanotechnology-enabled products as acknowledged by the participants of the online survey (no of respondents was equal to 15) and participants of the 2nd KEC (no of respondents in the Slido survey was equal to 24)

To better understand the regulatory needs and challenges from a stakeholder point of view, a survey and series of workshops were organised in the frame of the REFINE project. An online survey (July 2020 to March 2021) aimed to obtain quantitative feedback on the REFINE White Paper [1] and regulatory challenges for nanotechnology-enabled health products. The majority of participants were from academic institutions, but also from industry, notified bodies and governmental organisations/regulatory agencies. The respondents were equally representing the sectors of medical devices and medicinal products and acknowledged the relevance of all regulatory challenges identified in the white paper. The identification of regulatory information needs, lack of appropriate testing methods and the selection of the regulatory pathway, particularly relevant for borderline products, ranked the highest in the survey responses (Fig. 1). The methodological challenge was particularly acknowledged by representatives of academia. The application of the classification rule (rule 19) in combination with the European definition of nanomaterial is specific to the medical devices sector and was particularly recognised by representatives of this sector. Around 50% of the respondents judged that current regulatory guidance is not clear and not easy to access. They also highlighted the need for additional guidance documents, in particular for novel nanomaterials and for a stepwise comparability approach for follow-on (generic) medicinal products in order to reduce the uncertainty for product developers. Furthermore, the respondents suggested education and training programmes for healthcare professionals on handling of complex medicinal products and their generics. The development of computational approaches to evaluate clinical performance and safety was also proposed.

The identified regulatory challenges were also discussed at workshops and conferences with representatives of the medical field and other sectors, with the aim to identify similar challenges, knowledge gaps and regulatory questions across sectors and discuss potential common initiatives.

An example of such a workshop was the Gov4Nano Regulatory Risk Analysis Summit (RRAS) [10], which focused on the identification of nano-specific regulatory information needs across disciplines and regulatory domains. The challenges and aspects discussed at the RRAS were translated into research questions on the following topics: definition/harmonisation/equivalence, exposure, hazard and bridging the gap between science and regulation. Additionally, the summit focused on the process of identification of information needs and knowledge sharing. It was observed that there is no structured process in place to consult regulatory risk assessors to identify at least the most pressing trans-regulatory issues, to share knowledge or views and insights across regulatory domains or to construct and monitor trans-regulatory research roadmaps [11].

Two KECs were organised in the context of the REFINE project to discuss common regulatory challenges and synergies for nanotechnology-enabled products with representatives of different sectors including medical devices, industrial chemicals, food and cosmetics. In collaboration between Gov4Nano and REFINE, trans-sectorial regulatory issues and consequent research questions were identified and presented during the 2nd KEC [12]. The use of an interactive tool provided quantitative feedback from the participants. Among regulatory challenges that were common for different sectors, availability of suitable test methods and identification of quality attributes that have an impact on biological effects have received most recognition from the participants (Fig. 1).

In addition to the already identified regulatory questions and challenges, the workshop participants raised further needs (Box 2), such as the accessibility of usable and exchangeable characterisation data as well as to the comparability of test methods. Currently, the availability of a plethora of methods and instruments can create confusion, and it is unclear how data obtained with different methods can be related to each other and how the selected methods will be recognised by competent authorities. Another remaining challenge is the understanding of the entire lifecycle of the product, including pharmacovigilance and post-market surveillance, as well as environmental effects such as presence of nanomaterials in waste. Finally, the participants of the workshop stressed the need of an appropriate communication with consumers and patients on the benefits and risks of nanotechnology-enabled products. Public perception is crucial for the successful commercialisation of innovative technologies. Insufficient information can create a negative perception, which will reduce the success of consumer products and will hinder the uptake of highly effective health products into clinical applications.

## Addressing regulatory needs for nanotechnology-enabled health products in a changing political environment

The recognition of common challenges across sectors and proposed ways forward comes timely and is in line with objectives of the CSS [9]. In particular, the CSS initiative on *one substance one assessment* [13]*,* which aims to make safety assessment processes simpler and more transparent across regulatory domains, has outlined the future directions on how safety assessments in Europe should evolve. It will include a better coordination across regulatory frameworks and between agencies, the use of academic data, knowledge and data sharing platforms as well as coherent and possibly harmonised methodologies across different sectors. The exploitation of nanomaterials in different sectors would be an interesting case study to further explore the harmonisation of safety assessments across regulatory frameworks.

A better coordination across different sectors will help to reduce uncertainties, related to the assessment of innovative products based on nanotechnologies for which the selection of the regulatory path is challenging (the so-called borderline products) (Table 1). It has been shown that the assignment of certain innovative nanotechnology-enabled health products to the regulatory framework can trigger questions even if criteria of such assignment have been clearly defined. Only products with a pharmacological, immunological or metabolic primary mechanism of action or substances administered for the purpose of making a diagnosis are medicinal products. The challenge can be illustrated with some examples of different products based on iron oxide. A contrast agent, administered for the purpose of diagnosing an illness, e.g. cancer, will be classified as a medicinal product based on the definition. The same product, however, used by a surgeon to determine the boundaries for tumour excision and/or whether, or not, to remove the draining lymph nodes, has an intended purpose as a surgical aid rather than diagnosis and will thus be classified as a medical device. For medical devices, an additional challenge for products incorporating or consisting of nanomaterials is present by the application of classification rule 19 of the recently implemented Medical Device Regulation [14], which determines the conformity assessment procedure to be applied for such products, depending on their potential for internal exposure to the nanomaterial(s). Again, the rule turns out to trigger several questions of its interpretation when applied in practice.

**Fig. 2:**
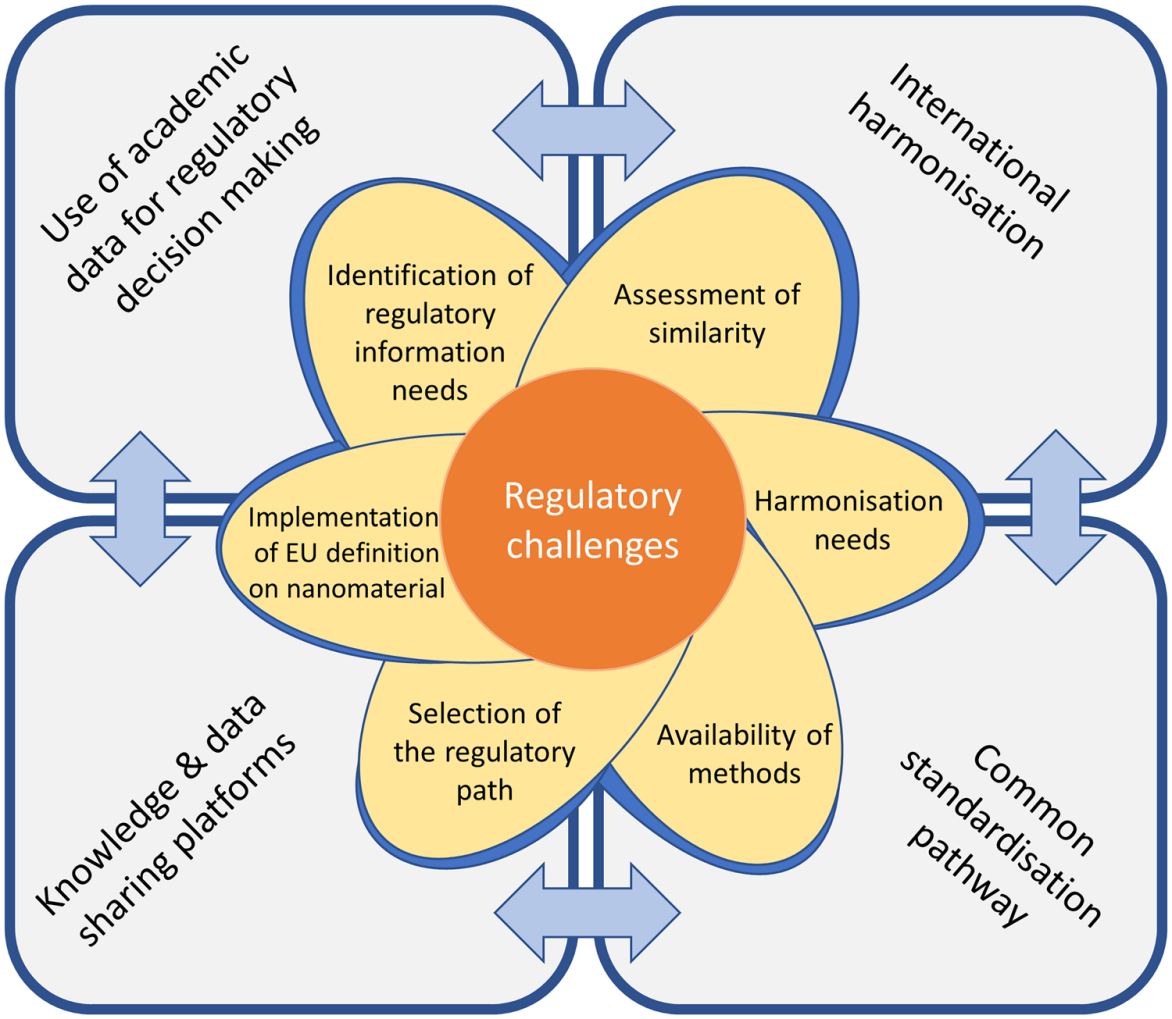
Future strategies and perspectives (background squares) in the regulatory science addressing regulatory challenges (flower leaves) for nanotechnology-enabled products

In the context of the upcoming regulatory initiatives and based on outcomes of the discussions with different stakeholders (regulatory scientists, product developers, risk assessors) and representatives of different communities, future strategies to overcome regulatory challenges for nanotechnology-enabled health products are proposed by the REFINE project (Fig. 2) and described in the sections below.

Box 2 Additional challenges and needs across different sectors using nanotechnology Need for accessibility of data, databases and data-sharing platforms

Comparability of test methods

Assessment of the entire lifecycle of nanotechnology products

Sufficient communication with consumers and patients

### Addressing knowledge gaps: use of academic data for regulatory decision-making

Despite a significant progress in the domain of nanobioscience and the improved understanding of the interaction of nanomaterials with the living systems, there are still some remaining knowledge gaps, which might hamper the regulatory decisions in relation to nanotechnology-enabled health products. In particular, the identification of critical quality attributes that have an impact on biological effects such as the modification of therapeutic effects or changes in the safety profile can be challenging for nanotechnology-based formulations. In fact, the continuous exploitation of nanotechnology leads to more complex formulations, where a whole set of different interlinked parameters will determine the biological effects. A better understanding of the impact of physicochemical properties on biological effects is also a key for the identification of crucial parameters that determine similarity (equivalence) of a follow-on product regarding an original product. Such knowledge is also of importance for emerging assessment tools in other sectors such as grouping and read across for nanotechnology-based products.

For innovative products where datasets derived from high quality studies are limited, the available academic data can assist in closing knowledge gaps and support an informed decision-making. The potential of academic research to address remaining questions with targeted, thoroughly prepared scientific studies was also recommended as a strategic goal by European Medicines Agency [15].

In any case, the use of academic data must overcome some hurdles before it can be taken up for regulatory decision-making. Currently, it is challenging and time-consuming to retrieve specific information from the scientific literature. More optimised tools to find and access the relevant scientific reports and data would enable the use of such knowledge in the regulatory environment. According to the FAIR principles,^7^ data should be findable, accessible, inter-operable and reusable. Currently, the availability of sufficiently large FAIR data sets is still scarce. In addition, more user-friendly automated tools for the systematic reviews of the scientific literature could speed up and optimise the process. Such tools are already being developed, but more effort is needed to make them more transparent, build trust and train potential users [16, 17].

Another caveat is often the need for criteria to judge the quality of the academic study, which should rely on robust methodologies and comply with high standards for the experimental approaches and reporting. Some standards for the quality criteria and reporting have already been proposed for nanotechnology-enabled products [18] or chemicals [19]^8^. The discussions are currently ongoing to develop such criteria in a broader perspective and enable the use of academic data in the regulatory environment [20]. Obviously, the sufficient quality of studies is related to the robustness and reliability of employed methods and their status of validation. The issue of standardisation and adoption of sufficient quality while using non-standardised methods is discussed in the Perspectives for coherent and harmonised method development and section.

Finally, an increased exchange between regulatory and research communities would benefit from training and education of academic researchers in the area of regulatory science and more explicit communication of regulatory needs to the research community.

### Harmonisation between geographical regions

A number of different fora are currently contributing to the exchange of knowledge among the international regulatory agencies aiming to increase scientific alignment in the assessment of novel and generic drugs (Table 2). The International Pharmaceutical Regulators Programme (IPRP) is an example of such a platform stimulating discussions on regulatory issues of mutual interest and enable cooperation. A working group on nanomedicine is particularly dedicated to nanotechnology-enabled health products [21] and is organising regular webinars. In one of such webinars, REFINE partners presented highlights of the REFINE project to more than 50 international regulatory scientists in a 1.5-h virtual session. In the sector of medical devices, the International Medical Devices Regulators Forum (IMDRF)^9^ aims to support regulatory harmonisation and convergence in the global perspective.

**Table 2:** Examples of international associations and initiatives for global harmonisation active in the field of nanotechnology-enabled health products

**Name of the organisation/platform**	**Description**
**European Federation of Pharmaceutical Sciences (EUFEP**S) (https://www.eufeps.org/nano-network.html)	EUFEPS Network on nanomedicine should focus on pharmaceutical and biomedical sciences and the diagnostics and therapeutic aspects of nanomedicine, primarily in cooperation with related nanotechnology fields such as optics, bioinformatics to achieve a wide range platform for pharmaceutical researchers in this field
**Global Bioequivalence Harmonisation Initiative (GBHI)** (https://pubmed.ncbi.nlm.nih.gov/34481066/)	The GBHI conferences are organised by EUFEPS in collaboration with American Association of Pharmaceutical Scientists (AAPS). The initiative aims to offer the most informative and up to date science and regulatory thinking of bioequivalence in global drug development to support the intended process of a scientific global harmonisation
**Lygature** (https://www.lygature.org/non-biological-complex-drugs-nbcd-working-group#project-update-1696)	The Non-Biological Complex Drugs (NBCD) Working Group was established to stimulate discussion of the safety and efficacy of NBCD innovation and follow-on products
**International Symposium on Scientific and Regulatory Advances in Biological and Non – Biological Complex Drugs (SRACD)** (http://sracd.hu/main-topics/)	A series of symposia addressing regulatory aspects of biological and non-biological complex drugs and the paradigm shift compared to the requirements for fully characterised small molecular drugs and the established formulations
**European Foundation for Clinical Nanomedicine (CLINAM**) (https://clinam.org/?page_id=102)	The recognition of the large future impact of nanoscience on medicine and the observed rapid advance of medical applications of nanoscience has been the main reasons for the creation of the CLINAM Foundation
**Global Coalition for Regulatory Science Research (GCRSR)** (https://www.fda.gov/about-fda/science-research-nctr/global-coalition-regulatory-science-research)	The Global Summit on Regulatory Science (GSRS) is an international conference for discussion of innovative technologies and partnerships to enhance translation of basic science into regulatory applications within the global context
**EDQM’s Working Party on Non-Biological Complexes (NBC)** (https://www.edqm.eu/en/groups-experts-and-working-parties)	The aim of the working party is the elaboration and revision of monographs on non-biological complexes (e.g. iron sucrose concentrated solution)

In July 2021, the US FDA and the EMA launched a pilot programme discussing the specific questions regarding complex generic drugs/hybrid products that are generally more challenging to develop with traditional bioequivalence methods. In this programme, the identified challenge on the assessment of “nanosimilars” [22–24] will now be addressed by the two major regulatory agencies. Product developers will benefit from this initiative and will be able to optimise their product development by avoiding unnecessary replication of testing when seeking for product authorisation for either the American or the European market. Interested product developers have the opportunity to join this pilot programme [25] and exchange their views on scientific issues with regulatory scientists during the development phase of complex generic drug/hybrid products.

### Knowledge and data sharing platforms

Since multiple sectors come across with similar challenges related to the complexity introduced by utilising nanomaterials, it is expected that solutions to address these challenges will also be similar. Knowledge sharing between the various domains is key to address the challenges in the optimal and most efficient way. Although there are large differences between the different regulatory domains, it will certainly be possible and fruitful to combine efforts and find common goals where knowledge sharing will create synergy. This was acknowledged by different stakeholders across sectors during the KECs [26]. To create added value by means of knowledge sharing, different expertise and perspectives are required. The knowledge sharing community must therefore maintain sufficient diversity to enable cross-disciplinary and innovative thinking. Domains to be included in the knowledge exchange are medicinal products, medical devices, cosmetics, chemicals/substances, food and biocides. Categories of stakeholders from the various domains that should be involved in order to include multiple perspectives are patients/consumers, healthcare professionals/end users, industry, regulators and academia. Scientific disciplines like physics, chemistry, biochemistry, immunology and toxicology in general should be stimulated to include case studies from multiple domains in their research programmes and also to join forces in multidisciplinary projects.

The organisation of existing knowledge into easily accessible knowledge and data sharing platforms would facilitate the exchange between different communities of stakeholders. Some examples already exist including the European Union Observatory for Nanomaterials (EUON)^10^, which provides information on nanomaterials existing on the European market and the related NanoData^11^ platform providing the access to research publications, projects and patents. The EU-funded NanoCommons project^12^ aims to integrate existing datasets on nanomaterials, toxicity and ecotoxicity following the FAIR principles. Similar platforms are to be developed under the new Horizon Europe partnership PARC (on chemicals risk assessment) [20] and the International Network Initiative on Safe and Sustainable Nanotechnologies (INISS-nano) [27].

Within the REFINE project, the concept of a knowledge hub (K-HUB) brings forward the need to connect, interact and exchange with the global multidisciplinary scientific, industrial and regulatory science communities. The creation of a multistakeholder functional and reliable exchange space should enable cross-fertilisation of data set, ease of access for read-across data and the creation of a multidisciplinary ecosystem, which will be more interactive than a repository archive. To achieve this goal, REFINE proposed and tested the establishment of a ResearchGate page^13^, as the tool selected for creating the K-HUB. ResearchGate is “the professional network for scientists and researchers. It helps researchers to connect and make it easy for them to share and access scientific output, knowledge, and expertise”. ResearchGate has the peculiarity of a social media-like platform, which allows a user to easily connect with colleagues, co-authors and experts in different fields to discuss the development, marketisation and regulatory approval process for nanotechnology-enabled medical products and devices. In addition, the modularity of the ResearchGate K-HUB allows interfacing and connection with the many other global initiatives focused on data sharing or repository knowledge. This can range from characterisation data on nanomaterials, to in vitro and in vivo safety data and in silico models for nanotechnology-enabled products.

Besides testing ResearchGate as a tool for cross-sectorial communication, the creation of a sustainable open-access repository archive to openly share knowledge as a community hub on scientific manuscripts, standard operating procedures, protocols, assays or data generated from the project was initiated. On this matter, REFINE adopted Zenodo^14^ as the open-access archiving solution since this has gained extensive visibility and access as a multistakeholder tool. All the REFINE public scientific and technical outcomes will be uploaded and shared under Zenodo REFINE community.

Clearly, the success of these platforms and their contribution to global multidisciplinary scientific, industrial and regulatory science knowledge sharing is heavily dependent of a sufficient number of users representing different communities and disciplines and actively sharing their knowledge and expertise.

### Perspectives for coherent and harmonised method development and standardisation

The use of any method for product assessment will require the regulatory acceptance of such a method by the regulatory bodies. Besides method-specific evaluations, this often includes a set of target values specified in the general validation guidelines provided by the different authorities. Within given fields, such as the medical area, regulatory bodies of various countries and continents have well harmonised these target values. It eases the formal approval process for new products across different countries, because identical or at least very similar statistical data evaluations can be submitted. However, the validation of each method used for producing the regulatory data is time and effort consuming.

The use of a standardised test method can ease regulatory requirements for the method validation and speed up the evaluation process. However, many currently available standardised methods were developed for small-molecule chemicals or pharmaceuticals and are not reliable when testing nanomaterials. Moreover, additional sophisticated methods are needed to evaluate nanomaterial-specific properties [3].

The analysis of methodological gaps for nanotechnology-enabled health products performed in the REFINE project identified the most pressing needs for method standardisation and development [28]. Some areas of methodological gaps are resulting from material-dependent applicability of the methods (Table 3), i.e. a given method might be compatible with a specific type of nanomaterials only. Therefore, any new method validation or standardisation requires the adequate testing with representative members of different product families (e.g. liposomes, polymers, iron oxides), without a reassurance, which new siblings of a given product family would fulfil the acceptance criteria of a regulatory/standardisation body. For example, the ASTM programme (Committee E.56 on Nanotechnology^15^) has included some very specific methods addressing sizing and composition of liposomal drug formulations and lipid nanoparticles, whereas ISO Technical Committee on Nanotechnologies^16^ has developed several standards addressing carbon nanotubes and graphene. However, the application of such specific methods to other nanotechnological platforms might be limited and would require additional testing. Areas with a huge innovation potential are facing the challenge of a continuously growing number of product families, which require additional verification of methods and their international standards.

**Table 3:** Types and examples of methodological gaps for nanotechnology-enabled health products

**Type of the gap**	**Complete gaps**	**Partial gaps (material-specific)**	**Partial gaps (method validation)**	**Partial gaps (sector of applicability)**
Description	No methods available	Existing standardised methods are applicable for certain types of nanomaterials only	Methods are available, but they are not standardised	Standardised methods are available in other sectors
Examples of methodological gaps	Quantification of large API such as nucleic acids	Determination of protein corona composition	Evaluation of the interaction with the immune system	Endotoxin measurement

*API* active pharmaceutical ingredient

**Fig. 3:**
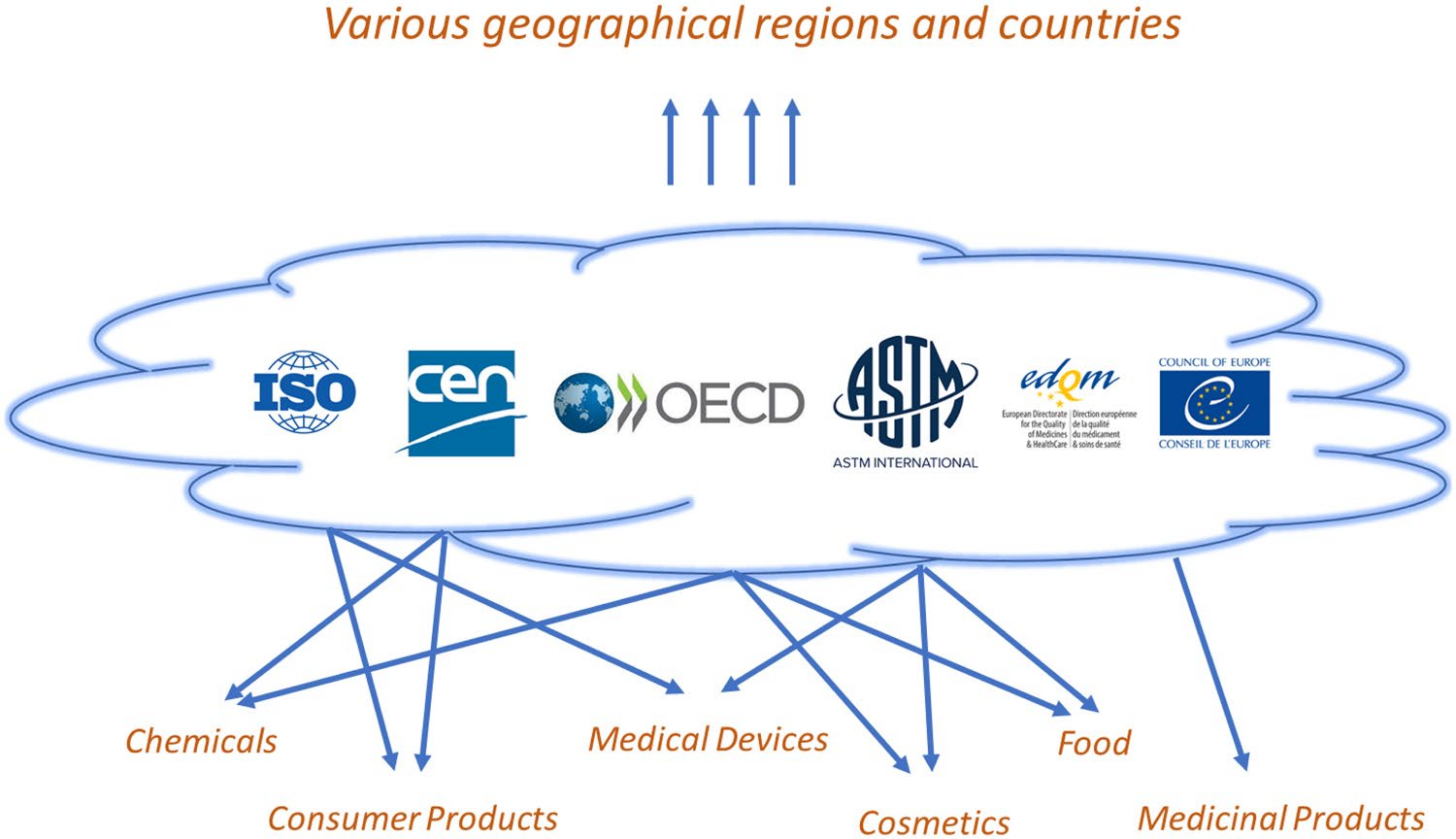
Main international standardisation bodies and their applicability to different regulatory frameworks and geographical regions

In addition, the applicability of standardised methods can differ depending on the regulatory framework, increasing the necessity of multiple standards across different fields. International standardisation organisations have either different subgroups, which focus their attention to a given area, or national, respectively, international standardisation organisations themselves focus largely on one field, such as consumer products (Fig. 3). The organisation, membership rules and standardisation workflow can significantly differ from one standardisation body to another. Whereas most of international standardisation organisation (e.g. ISO, OECD) has a membership arrangement, which is based on country representations, ASTM International applies an open membership policy with attempting to balance representation of government regulators, of industrial and of academic based members. The recognition of standards can vary also according to geographical region, requiring, e.g. in Europe the CEN and ISO standards, whereas other standards can be preferred in non-European countries.

The described situation suggests that the already thin resources for the international standardisation of newly developed methods will become even more scarce. Since nanotechnology-enabled products are used across many regulatory frameworks, a closer collaboration and coordination between the different international standardisation bodies and their mutual recognition agreements would be beneficial to avoid duplication of work and focus on common priorities in this innovative and accordingly strongly growing field.

Alternatively, possibilities to use non-standardised methods of sufficient quality to produce regulatory data could be investigated to reduce the necessity of the standardisation of the continuously growing number of new methods. In this case, mutually accepted quality criteria for new methods should be developed by representatives of different communities and sectors.

## Sustainability of REFINE tools, approaches and methods: perspectives

The new Chemical Strategy for Sustainability highlights the need for a trans-sectorial alignment of risk assessments of substances while taking into account the specificities of each sector (*one substance one assessment 1S1A)* [13]. The REFINE project could serve as an example demonstrating how some objectives of the initiative 1S1A could be achieved. Following the identification of regulatory challenges including methodological needs and gaps related to the assessment of nanotechnology-based health products, suitable test methods and approaches were proposed and tested within the REFINE project. They included several in vitro test methods and models for the evaluation of safety and in silico tools for the prediction of pharmacokinetic behaviour of nanotechnology-enabled products. Through the series of inter-laboratory comparison studies, the standard operation protocols were verified and harmonised as a basis for further standardisation.

In addition, a decision support system (REFINE DSS^17^) has been developed to guide the physicochemical characterisation and immunotoxicity assessment of medical products and devices based on nanotechnology.

These experimental approaches and results were presented to the IPRP working group on nanomedicines, gathering regulatory scientists from worldwide regulatory agencies. It was an exceptional opportunity to bring the REFINE outcomes and perspectives to the attention of this international group of regulators, making them aware of new methodological developments and promoting future use of REFINE methods and tools.

The availability and sustainability of obtained outcomes and knowledge for different communities and stakeholders including product developers, regulators, policy makers and industry were a clear interest of the consortium. Besides IT-based solutions to reach and bridge different communities (Knowledge Hub, Repository), first steps were undertaken to initiate connections with highly innovative and industry-focused Open Innovation Test Bed (OITB) clusters such as MDOT^18^ and Safe-N-Medtech^19^ as well as the SUNSHINE^20^ project to evaluate opportunities to transfer methods and approaches developed in REFINE. In fact, such transfer would greatly contribute to the enlarged catalogues of services provided to the medical technology industrial environments. In particular, the DSS tool as well as some in vitro methods for the characterisation of biological responses were of special interest to the OITBs and might be further developed in these projects. In the meantime, the data obtained with novel methods and the SOPs developed in REFINE will also be made available to the research community through scientific peer-reviewed publications and open-access platforms such as ResearchGate and Zenodo.

Another important approach was to discuss the outcomes of the REFINE work with different stakeholder groups and communities. The Knowledge Exchange Conferences and especially the exchange with highly complementary project Gov4Nano turned out to be a very fruitful connection and collaboration. Several discussions around the need for a trans-sectorial alignment of risk assessments of products resulted in the back-to-back organisation of the 2nd Regulatory Risk Assessment Summit (Gov4Nano) and the 3rd Knowledge Exchange Conference (REFINE) in January 2022. As a highlight, a joint session connecting both events was held to illustrate common and sector-specific challenges. The joint session intended to demonstrate the need for trans-sectorial strategies, to share common challenges and to provide future perspectives to facilitate harmonised regulation of products based on nanotechnology. Thus, the collaboration with Gov4Nano represents an excellent example of community bridging allowing the sustainability of the generated outcome and perspectives obtained in the project.

**Fig. 4:**
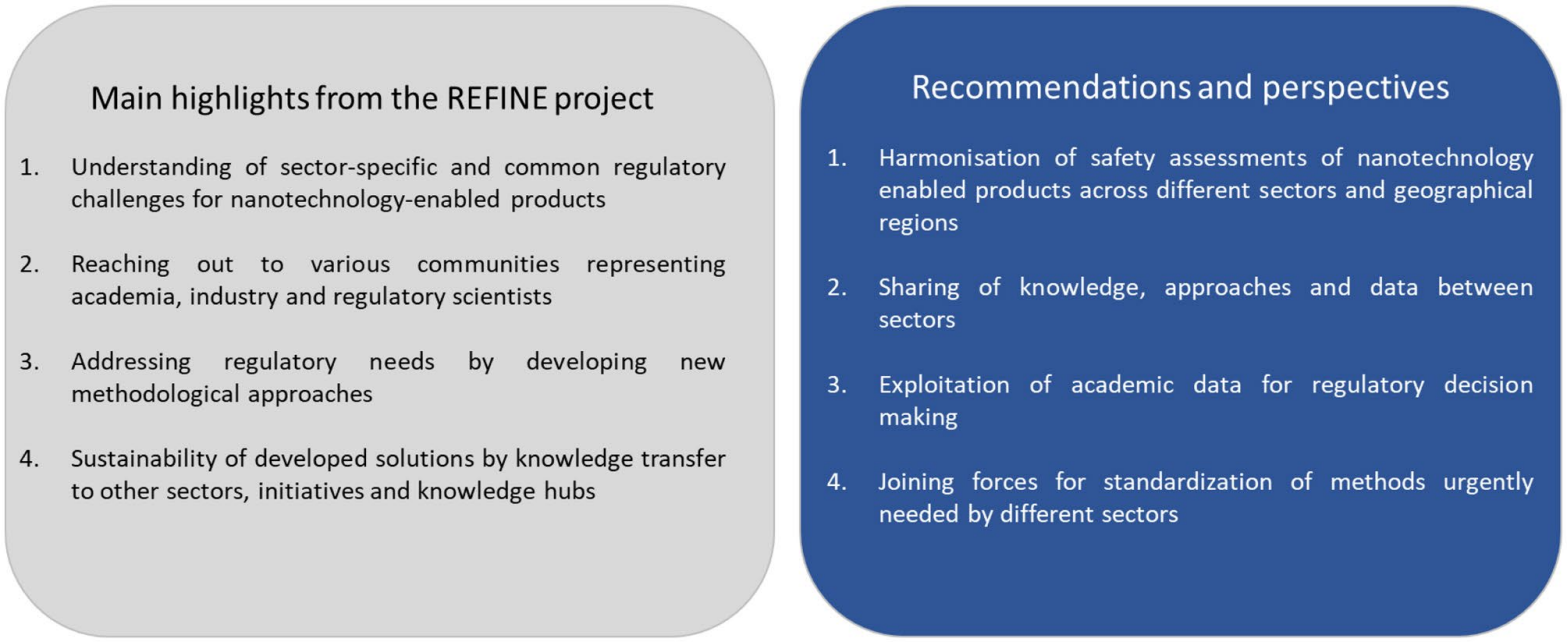
Main highlights and recommendations from the REFINE project

The heritage of REFINE is certainly found in the outstanding integration and addressing of identified regulatory challenges in the experimental development of new in vitro methods for nanotechnology-enabled health products. In summary, the REFINE project can serve as a blueprint for structural and straightforward development of regulatory science frameworks in different sectors and approaches to interact and exchange information and experience within multi-sectorial community. The main REFINE outcomes and the resulting recommendations of the project are summarised in Fig. 4.

## Footnotes

1 International Pharmaceutical Regulators Programme (IPRP); https://www.iprp.global/working-group/nanomedicines

2 EU-Innovation network; https://www.hma.eu/495.html

3 EC New Technologies WG for medical devices; https://ec.europa.eu/health/md_dialogue/mdcg_working_groups_en#seven

4 Gov4Nano project; https://www.gov4nano.eu

5 REFINE Second Knowledge Exchange Conference; http://refine-nanomed.eu/refine-second-knowledge-exchange-conference/

6 Regulatory Risk Analysis Summit 2019; https://www.gov4nano.eu/event/regulatory-risk-analysis-summit-2019/

7 FAIR principles, GO FAIR; https://www.go-fair.org/fair-principles/

8 Science in Risk Assessment and Policy; http://www.scirap.org/

9 International Medical Devices Regulators Forum; https://www.imdrf.org/

10 European Union Observatory for Nanomaterials; https://euon.echa.europa.eu/

11 NanoData platform; https://nanodata.echa.europa.eu/

12 NanoCommons project, https://www.nanocommons.eu/

13 REFINE project on the ResearchGate: https://www.researchgate.net/project/REFINE-Regulatory-Science-Framework-for-Nanobiomaterial-based-Medical-Products-and-Devices

14 REFINE project on Zenodo; https://zenodo.org/communities/refine_eu_h2020_project

15 ASTM International/Committee E56 on Nanotechnology. List of standards for physical and chemical characterisation available at: https://www.astm.org/get-involved/technical-committees/committee-e56/subcommittee-e56/jurisdiction-e5602

16 ISO Technical Committee on Nanotechnologies. List of active standards available at https://www.iso.org/committee/381983/x/catalogue/p/1/u/0/w/0/d/0

17 REFINE Decision Support System; https://www.refinedss.eu/login

18 Medical Device Obligations Taskforce (MDOT); https://cordis.europa.eu/project/id/814654

19 Safe-N-Medtech project: Safety Testing In The Life Cycle Of Nanotechnology-Enabled Medical Technologies For Health; https://cordis.europa.eu/project/id/814607; https://safenmt.eu/

20 Safe and sustainable by design strategies for high performance multi-component nanomaterials (SUNSHINE); https://cordis.europa.eu/project/id/952924
